# Conversation focused aphasia therapy: investigating the adoption of strategies by people with agrammatism

**DOI:** 10.1080/02687038.2014.881459

**Published:** 2014-02-07

**Authors:** Suzanne Beeke, Firle Beckley, Fiona Johnson, Claudia Heilemann, Susan Edwards, Jane Maxim, Wendy Best

**Affiliations:** ^a^ Division of Psychology and Language Sciences, University College London, London, UK; ^b^ Guys & St Thomas’ NHS Foundation Trust, London, UK; ^c^ Psychology and Clinical Language Sciences, University of Reading, Reading, UK

**Keywords:** aphasia, conversation therapy, communication strategies, agrammatism, behaviour change

## Abstract

*Background*: A recent review of interaction (or conversation)-focused therapy highlighted the potential of programmes targeting the person with aphasia (PWA) directly. However, it noted the key limitations of current work in this field to be a reliance on single case analyses and qualitative evidence of change, a situation that is not unusual when a complex behavioural intervention is in the early stages of development and evaluation.

*Aims*: This article aims to evaluate an intervention that targeted a PWA and their conversation partner (CP), a dyad, as equals in a novel conversation therapy for agrammatic aphasia, using both quantitative and qualitative evidence of change. The intervention aimed to increase the insight of a dyad into facilitator and barrier conversation behaviours, to increase the understanding of the effect of agrammatism on communication, and to support each speaker to choose three strategies to work on in therapy to increase mutual understanding and enhance conversation.

*Methods & Procedures*: Quantitative and qualitative methods are used to analyse multiple pre-therapy and follow up assessments of conversation for two dyads.

*Outcomes & Results*: Results show that one person with severe and chronic agrammatic aphasia was able to select and practise strategies that led to qualitative and quantitative changes in his post-therapy conversations. The other PWA showed a numerical increase in one of his three strategies post therapy, but no significant quantitative change. Although both CPs significantly reduced barrier behaviours in their post-therapy conversations, neither showed a significant increase in the strategies they chose to work on. For one CP, there was qualitative evidence of the use of different turn types.

*Conclusions*: Individually tailored input from a speech and language therapist can assist some people with chronic agrammatism to develop conversational strategies that enhance communication. Outcomes are influenced by the severity and extent of language deficits affecting, for example, single word writing. In terms of behaviour change for CPs, it appears that it may be easier to reduce barrier behaviours rather than to increase the use of facilitatory strategies. The results have implications for collaborative goal setting with clients undergoing conversation therapy.

## Background

Conversation partner (CP) training programmes have become increasingly popular over the last decade (e.g., see Kagan, 1998; Lock, Wilkinson, & Bryan, 2001; McVicker, Parr, Pound, & Duchan, 2009; see Wilkinson, 2010 for a review). A systematic review by Simmons-Mackie, Raymer, Armstrong, Holland, and Cherney (2010) has concluded that conversation training is effective at improving the communication of a CP, and having a trained CP is probably effective in also improving the participation in conversation of a person with chronic aphasia. One set of studies has focused on training CPs in groups without the person with aphasia (PWA) being present (Booth & Swabey, 1999; Kagan, Black, Duchan, Simmons-Mackie, & Square, 2001; McVicker et al., 2009), while another has trained CPs and PWAs within dyads (Burch, Wilkinson, & Lock, 2002; Cunningham & Ward, 2003; Lock et al., 2001; Sorin-Peters, 2004), but in all these studies, the focus is primarily on effecting change to the CP’s behaviour in order to indirectly influence the PWA’s conversation.

In a review of conversation therapy studies, Wilkinson and Wielaert (2012) highlight not only the success of CP training, but also the potential of similar programmes that train a PWA directly. For example, Wilkinson, Lock, Bryan, and Sage (2011) successfully taught a PWA to use topic alerters as a new method of initiating topics, and Wilkinson, Bryan, Lock, and Sage (2010) employed direct training of a PWA to increase the development of topics. In addition, Fox, Armstrong, and Boles (2009), employing procedures used by Boles (1998) and Boles and Lewis (2003), noted improved PWA participation in conversation after the training. Specifically the PWA initiated more topics, asked more questions and slowed rate of speech. These changes were verified by PWA and CP report and observation measures. Thus far, results of PWA conversation training have been encouraging. However, Wilkinson and Wielaert (2012) note a reliance on single case analyses and qualitative evidence of change. As the authors point out, although this is not unusual when a complex behavioural intervention is in the early stages of development and evaluation, it limits the robustness of the evidence base.

This article reports on a tailored conversation therapy programme that was designed to allow direct work with a PWA and with their CP (see Beckley et al., 2013; Beeke, Maxim, Best, & Cooper, 2011). The therapy aimed to raise insight into the effects of agrammatism on conversation and teach strategies to allow (i) a PWA to produce more complete, and thus successful, turns at talk, thereby increasing the likelihood of mutual understanding, and (ii) a CP to modify their responses to PWA turns and, thus, enhance their partner’s chance of communicating more effectively. Participants were facilitated to choose three strategies to work on from a restricted set of suggestions. Each strategy had an interactional focus and was based on CA research into successful turn construction behaviours of individuals with agrammatism (Beeke, Wilkinson, & Maxim, 2003, 2007) and CP strategies that have been found to aid the flow of conversation (Lock et al., 2001). PWA strategies included the use of a keyword (to identify what is being talked about) and the integration of writing, drawing, use of props, gesture, or facial expression into a turn. Thus, the therapy was not impairment-focused with the objective of reducing agrammatic output, instead it targeted both speakers, facilitating the development of a communicative conversation style in spite of agrammatism.

## Aims

The aims of the study were to use a mixed methods approach to evaluate a conversation-focused intervention that trained both the CP and the PWA, in two dyads where one speaker has agrammatic aphasia. This article presents quantitative outcomes of conversation change and qualitative insights into why behavioural change in conversation may or may not have occurred for the PWA and CP, using conversation Analysis (CA). We present two illustrative case studies from a larger study, chosen for the similarity of the barrier conversation behaviour used by both CPs (test questions, defined below) and the extent to which it dominated pre-therapy conversations. An analysis of all eight dyads is in preparation.

## Methods and procedures

**Figure 1.**
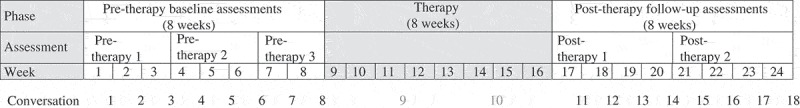
Study design.

Each case study involved a PWA and their chosen CP in 6 months of intervention, subdivided into three phases of 8 weeks each: (a) pre-therapy baseline assessment; (b) therapy; and (c) post-therapy follow up assessment. Figure 1 illustrates the design.

### Assessments

A dyad participated in 8 weeks of pre-therapy and 8 weeks of post-therapy assessment, which took place at home, once a week for around 1.5 hr. Assessment of conversation involved video recording eight pre- and eight post-therapy samples, labelled C1–8 and C11–18, respectively, and two conversations during therapy (C9 and C10, not analysed here), each of approximately 20 min in length, totalling around 6 hr of recordings per dyad. To do this, a dyad was trained in how to operate a digital video camera. The research speech and language therapist (SLT) was not present when the recordings were made. Dyads were advised to record at a time when they would normally sit down for a chat, to catch up on events and news, for example; there were no suggested topics. Assessment of language impairment, activity, and participation was split into three pre-therapy and two post-therapy assessment baselines (see Figure 1) and consisted of a battery of tests and interviews and a test of cognitive flexibility (see Table 1). Some tests were repeated once at each baseline (i.e., three times before therapy and twice after therapy, see Figure 1) in order to capture any change, and maintenance of change, post therapy. These assessments are referred to as repeated measures in Table 1. However, other tests were administered to gain an overview of performance and, thus, were only carried out once before and once after therapy (referred to as profile assessments in Table 1). In this article, we report on pre-therapy language assessment results to give the reader a profile of each participant’s aphasia. Thus, scores obtained from measures repeated before therapy have been averaged.

**Table 1.** Assessment battery.

Repeated measures
**Object and action naming battery** (Druks & Masterson, 2000)
20 items: 10 nouns and 10 verbs
**Psycholinguistic assessments of language processing in aphasia** (PALPA, Kay et al., 1992)
PALPA 53 written single words (30 items)
**Comprehensive aphasia test** (CAT, Swinburn et al., 2004)
Repetition of digit strings
Comprehension of written sentences
Spoken picture description
**Verb and sentence test** (VAST, Bastiaanse, Edwards, & Rispens, 2002)
Sentence production
**Dinner party narrative** (Fletcher & Birt, 1983)
**Communication disability profile** (CDP, Byng & Swinburn, 2006)
**Conversation analysis profile of people with aphasia** (CAPPA, Whitworth, Perkins, & Lesser, 1997)
Profile measures
**Pyramids and palm trees test** (Howard & Patterson, 1992)
3-picture version
**Psycholinguistic assessments of language processing in aphasia** (PALPA, Kay et al., 1992)
PALPA 4 minimal pair discrimination
PALPA 47 spoken word picture match
**Verb and sentence test** (VAST, Bastiaanse et al., 2002)
Comprehension of spoken sentences
**The Hayling and Brixton tests of Dysexecutive Syndrome** (Burgess & Shallice, 1997)
Brixton spatial anticipation test

### The *Better Conversations with Aphasia* therapy programme

Therapy sessions took place at the participants’ home once a week for 8 weeks, each lasting around 1.5 hr. The therapy, designed specifically for people with agrammatic aphasia, was adapted from SPPARC (Lock et al., 2001) but with two significant differences: (i) it introduced participants to agrammatism and its specific effects on conversation, such as difficulties with “building” a turn; and (ii) it aimed to change the conversational behaviours of both the PWA and CP, via direct work with each on strategy use in conversation. Appendix A provides a list of the sessions, grouped according to their main aims (developing insight, choosing strategies, practising strategy use). Following the SPPARC ethos (also called “interaction therapy”, see Wilkinson, 2010), during sessions 1, 2, and 3, a dyad viewed short video clips from pre-therapy conversations to increase their insight into key features (both positive and negative) of their interactions (referred to as facilitators and barriers) and to identify what they could have done differently during problematic exchanges. Having raised a dyad’s insight in this way, the SLT then facilitated a joint goal setting process, with the outcome that both the PWA (in session 4) and the CP (in session 5) were encouraged to choose three strategies each that they wished to practise in therapy to reduce barriers and aid the flow of conversations. In session 6, both the PWA and the CP chose strategies to aid topic setting and development, as detailed in SPPARC. It should be noted that because of the extensive use of videos of the dyad, although the main aim of sessions 4, 5, and 6 was to facilitate strategy choice, these sessions also continued to develop insight. The therapy was also distinctive in that it included two sessions (7 and 8) during which the dyad held “practise” conversations and received feedback from the SLT on strategy use, both as the practise conversation was happening (online coaching) and after it ended (this included watching it back on video). For further details of session aims and techniques, see Beckley et al. (2013). To access the therapy programme itself, visit Better Conversations with Aphasia, a free e-learning resource (https://extend.ucl.ac.uk).

### Quantitative and qualitative analysis of conversation samples

The conversation samples were analysed quantitatively by counting instances of facilitators and barriers in twelve 5-min video samples, taken from six pre- and six post-therapy conversations (from a total of eight pre- and eight post-therapy samples), and applying a statistical test to look for significant change. Sample selection was motivated by the wish to minimise participant awareness of being part of the study, and particularly of being video recorded. Thus, we discarded C1, the very first conversation that participants recorded before therapy, and C18, the final post-therapy sample. The remaining seven pre- and seven post-therapy samples were felt to be more ecologically valid. Of these 14 samples, to date we have counts for 12 (C2–4 and C6–8 pre-therapy, and C11–14 and C16 and C17 post-therapy), and this analysis is reported here. Where a conversation sample was 10 min in length or longer, the first 5 min was discarded, with the second 5 min selected for analysis to maximise ecological validity. If a sample was less than 10 min in length, the final 5 min was selected for analysis.

The counts of facilitators and barriers reported here are the work of six Masters students of speech and language therapy at University College London, who completed their theses with the project team between 2010 and 2012. Prior to rating, students (all of whom had completed a minimum of 2.5 hr of basic CA training as part of their degree course) received 6.5 hr of group training split across four workshops, covering the aims and design of the project, how to produce a transcript of a sample (one turn to a line) prior to rating (a detailed transcript was not deemed necessary, but important non-verbal communication was included), and how to rate a sample for barriers and facilitators. Student raters were given written guidelines that defined each barrier and facilitator and gave real-data examples, and workshops included a discussion of these definitions followed by group rating practice, with conversation samples taken from two people with agrammatic aphasia who were not part of the project. During the rating process, students attended a further two group meetings at which they could discuss any difficulties with rating and categorising aspects of conversation. They also had access to a group e-mail via which they could post queries to their fellow students and the project team. Each student was allocated between two and eight samples to rate (with an equal number taken from pre- and post-therapy samples), depending on the scope of their project. All were blind at the time of rating as to the point of collection for all samples. In some cases, it was possible to ask two students to independently rate the same sample and to agree counts for individual facilitators and barriers. Agreement was defined as both students applying the same rating to a specific turn; consensus agreement techniques were not used. Where available, we report counts of facilitators/barriers agreed by two students, even if this is zero. Otherwise, counts by an individual student are reported.

The counts constitute non-parametric frequency data across occasions. The Poisson distribution can be applied where there are a large number of possible events which are rare. A weighted Poisson trend test for frequencies, derived from a Jonckheere Trend Test, was applied to identify whether there was a significant change in these counts after therapy (David Howard, May 2011, personal communication). This enabled us to test the hypothesis that the post-therapy counts differed significantly from the pre-therapy counts. To test this, all pre-therapy conversations were weighted the same and all post-therapy conversations were weighted the same. We did not explore other possible hypotheses, such as gradual change over the course of the study. It should be noted that, if the number of observations in any condition is less than 5, the *z* score approximation may not be very accurate. Because of the directional hypotheses for change, we employed one-tailed tests.

In addition, the methods and published findings of CA were applied to conversational extracts to illustrate the impact on conversation of a reduction in CP barrier behaviours and an increase in PWA strategy use and the interconnected nature of such changes. Three of the four extracts analysed here (Extracts 2, 3, and 4) were originally identified, transcribed using CA conventions and analysed by three of the six Masters students. This formed part of the qualitative data presented in their theses alongside the conversation ratings. They performed this work after producing the ratings, when they had been unblinded as to sample collection date. Their transcripts and analyses have been refined and extended by the first author.

### Participants

Two dyads are reported here. The first is Graham, a right-handed male British-English speaker with severe non-fluent aphasia, and his partner, Alex (throughout this article, all names are pseudonyms). Graham was 63 years old when recruited to the project, having had a left middle cerebral artery infarct 5 years before, affecting the fronto-temporal cortex and parts of the parietal lobe. After the stroke, Graham was in intensive care for 4 weeks. He then received 12 weeks of National Health Service (NHS) inpatient rehabilitation followed by a further 6 sessions of community speech and language therapy. After this he had private SLT for 3 years, twice a week for the first 6 months, weekly thereafter. Graham left school at 16 years, trained and worked as a nurse, and latterly worked as a nurse manager in several hospitals. Alex is a retired accident and emergency nurse and was in his early 60s when recruited to the project.

**Figure 2.**
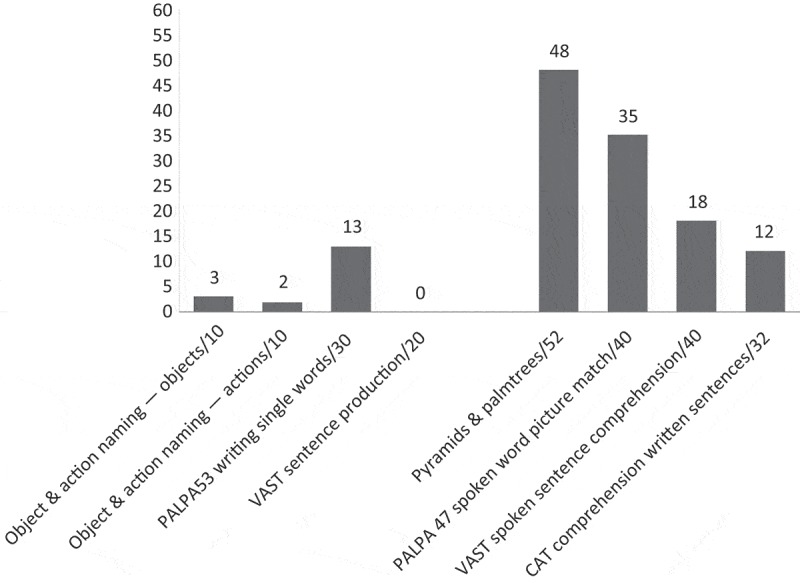
Pre-therapy language profile: Graham.

As Figure 2 shows, although non-verbal semantics was largely intact and single word comprehension was mildly impaired, Graham’s comprehension was severely impaired for spoken and written sentences. He was severely impaired on spoken word retrieval and sentence production. His ability to write single words was less impaired than his spoken naming. As this sample from the CAT picture description (Swinburn, Porter, & Howard, 2004) shows, Graham’s connected speech is severely affected by his aphasia and also a moderate to severe verbal dyspraxia (numbers in brackets indicate pauses in seconds):

“a cat (2) and (2 sylls) and the (2 sylls) (1) and a then a another one and (8) a (1 syll) and (SLT: that’s it?) yes”

His test profile is suggestive of severe agrammatic aphasia.

During therapy, after viewing video footage of their conversations and discussing facilitators and barriers with the SLT, Graham chose to work on the following strategies: writing and drawing; mime; keyword. Alex chose to work on: “let the conversation continue (for further clues/so Graham can use strategies)”; “carry on if you have understood (it does not need to be perfect)”; “comment” on Graham’s turn. A key conversational barrier discussed with the dyad was Alex’s use of test questions. A test (or “known answer”) question is defined as one to which the questioner already knows the answer and was first outlined in the context of formal talk in the classroom (McHoul, 1978; Searle, 1969).

The second dyad is Stuart, a right-handed male British-English speaker with severe non-fluent aphasia, and his wife, Pamela. Stuart was 57 years old when recruited to the project, having had a left cerebrovascular accident 10 months before (no scan results were available). After the stroke, Stuart received NHS inpatient rehabilitation for an unknown length of time before having 12 weeks of community SLT. Stuart left school at 16 years, and, prior to his stroke, he worked as a self employed van driver. Pamela is a school dinner lady and was in her mid-50s when she and Stuart joined the study. They live with their son, Graham, who was 12 years old at the time of their involvement in the project. Some of the conversations recorded by Pamela and Stuart include Graham (though he is not visible on camera).

**Figure 3.**
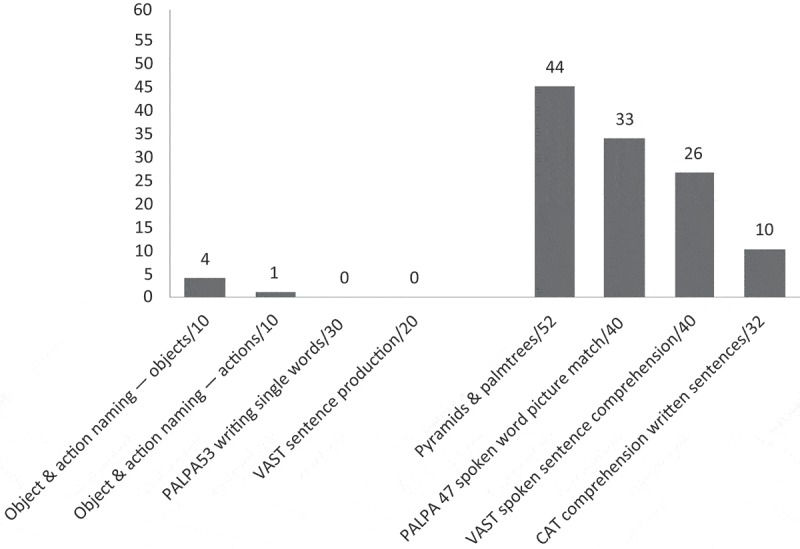
Pre-therapy language profile: Stuart.

As Figure 3 shows, Stuart showed a mild impairment of non-verbal semantics, a mild problem understanding spoken words, but a moderate difficulty understanding spoken sentences. He was severely impaired on a test of understanding written sentences. Production of single words and sentences, spoken and written, was severely impaired. As this sample from the CAT picture description (Swinburn et al., 2004) shows, Stuart’s connected speech is severely affected by his aphasia (numbers in brackets indicate pauses in seconds):

“yeah yeah picture and (1) book (1) book umm tree (1) tree uh uh uh and ((taps with fingers)) (3) (1 syll) (10) picture (4) and book and (3) book and (SLT: it’s alright) (9) book and right (2) know what I mean, d’you know what I mean, ooh oh (SLT: anything else) book and (2) book, yes (6) book (3 sylls) look look no one two three four five (1) book book book and woman woman woman (3 sylls) and run run run run (3 sylls) (10) (SLT: yes) ((PWA writes)) (4) miaow miaow miaow miaow”

His test profile is suggestive of severe agrammatic aphasia.

During therapy, after viewing video footage of their conversations and discussing facilitators and barriers with the SLT, Stuart chose to work on the following strategies: writing and drawing; gesture; keyword. Pamela chose to work on: “let the conversation continue (for further for clues/so Stuart can use strategies)”; “carry on if you have understood (it does not need to be perfect)”; “paraphrase” what you think Stuart said/meant. In common with Graham and Alex, a key conversational barrier discussed with this dyad was Pamela’s use of test questions.

## Results

We predicted that there would be an increase in behaviours targeted as helpful in conversation (facilitators) and a decrease in behaviours deemed unhelpful (barriers). Here we present results of statistical analysis of strategies chosen by the PWA and by the CP (deemed facilitatory behaviours), and CP barriers, for six pre- and six post-therapy conversation samples, to identify whether therapy had a significant effect on conversation. This section is followed by a qualitative analysis of conversation extracts that explores how the turns taken by the PWA and CP changed after therapy.

### Quantitative results: conversation change after therapy

#### Graham and Alex

Table 2 summarises the effects of therapy on targeted facilitators and barriers for Graham and Alex.

**Table 2.** Graham and Alex—Counts of strategies and barriers in pre- and post-therapy conversation samples.

Sample	Pre-therapy						Total pre-therapy (mean per sample)	Post-therapy						Total post-therapy (mean per sample)
	C2	C3	C4	C6	C7	C8		C11	C12	C13	C14	C16	C17	
PWA chosen strategies														
Writing (and drawing†)	0*	0	0*	0	0	0	0 (0)	0	0	0	0*	7	1	8 (1.33)
Mime	0*	0	0*	1	0*	0	1 (0.167)	3	3	0	0*	0	0	6 (1)
Keyword	3*	0	2*	1	0*	3	9 (1.5)	8	5*	5	2*	4	2*	26 (4.33)
CP chosen strategies														
Let conversation continue	0	0	0*	0	4	7	11 (1.83)	2	2	1	0*	5	0	10 (1.67)
Carry on if have understood	0	2	1*	3	3	3	12 (2)	1	3	3	2*	1	0	10 (1.67)
Comment	1*	1	0*	4	9	12	27 (4.5)	3	7	5	2*	5	5	27 (4.5)
CP targeted barrier														
Test question	7*	1	0*	10	1	16	35 (5.83)	2	1	0	0*	2	0*	5 (0.83)

† Although writing and drawing were presented as *one* strategy choice during therapy, Graham did not use any drawing in the pre- and post-therapy samples analysed here; these counts represent writing.

* Counts with an asterisk were agreed upon by two independent raters; all other counts were generated by a single rater. All raters were blind to sample collection dates.

For Graham, the PWA, strategies that he chose to work on in therapy all increased significantly in post-therapy samples: Poisson trend for frequencies (1-tailed), writing (*z* = 2.83, *p* < .01); mime (*z* = 1.89, *p* < .05); keyword (*z* = 2.87, *p* < .01). (Note that there were no counts of drawing in pre- or post-therapy samples, so the result applies only to writing). As Table 2 shows, there were no counts of writing in *pre-*therapy samples, thus the result for writing appears to reflect adoption of a new strategy. However, the counts for mime and keywords reveal that Graham was making some use of both before therapy, so the results reflect increased use of these behaviours, rather than adoption of new strategies. For Alex, the CP, there was no significant effect of therapy on strategies that he chose to work on: Poisson trend for frequencies (1-tailed), “let the conversation continue” (*z* = −0.22, ns), “carry on if you have understood” (*z* = −0.43, ns), “comment” (*z* = 0.00, ns). For each strategy, the numerical data for pre-therapy and post-therapy total counts are stable, as are the means. However, there was a significant result for test questions, a barrier behaviour in Alex’s conversations that was targeted for reduction: Poisson trend for frequencies (1-tailed) test questions (*z* = −4.74, *p* < .0001). Thus, for Alex, behaviour change as a result of therapy constituted the reduction (almost the eradication) of a barrier conversation behaviour (asking test questions), but there was no corresponding increase in his three chosen strategies, which remained at pre-therapy levels.

#### Stuart and Pamela

Table 3 summarises the effects of therapy on targeted facilitators and barriers for Stuart and Pamela.

**Table 3.** Stuart and Pamela—Counts of strategies and barriers in pre- and post-therapy conversation samples.

Sample	Pre-therapy						Total pre-therapy(mean per sample)	Post-therapy						Total post-therapy(mean per sample)
	C2	C3	C4	C5§#	C6#	C7#		C11#	C12#	C13#	C14#	C16#	C17#	
PWA chosen strategies														
Writing (and drawing†)	1*	0	3*	1	3	2*	10 (1.67)	0	1*	3	0*	3	1*	8 (1.33)
Gesture	6*	17	6*	9	5	2*	45 (7.5)	5	3*	10	0*	9	4*	31 (5.167)
Keyword	2*	5	1*	2	4	1*	15 (2.5)	5	1*	8	3*	4	2*	23 (3.833)
CP chosen strategies														
Let conversation continue	1	2	2*	0	3	2	10 (1.67)	2	2	4	0*	4	2	14 (2.33)
Carry on if have understood	1	1	1*	1	0	0	4 (0.67)	0	0	1	0*	1	0	2 (0.33)
Paraphrase	0*	3	0*	3	4	0*	10 (1.67)	1	1*	4	0*	1	0*	7 (1.167)
CP targeted barrier														
Test question	14*	0	17*	7	7	4*	49 (8.167)	0	0*	0	0*	2	2*	4 (0.67)

§ Stuart and Pamela did not record C8; therefore, C5 has been analysed in its place.

# Samples in which Stuart’s and Pamela’s 12-year-old son is present.

† Although writing and drawing were presented as one strategy choice during therapy, Stuart did not use any drawing in the pre- and post-therapy samples analysed here; these counts represent writing.

* Counts with an asterisk were agreed upon by two independent raters; all other counts were generated by a single rater. All raters were blind to sample collection dates.

For both Stuart (the PWA) and Pamela (the CP), there was no significant effect of therapy on strategies that they chose to work on: Poisson trend for frequencies (1-tailed), Stuart: writing, (*z* = −0.47, ns), gesture (*z* = −1.61, ns), keyword (*z* = 1.30, ns); Pamela: “let the conversation continue” (*z* = 0.82, ns), “carry on if you have understood” (*z* = −0.82, ns), “paraphrase” (*z* = −0.73, ns). Note that there were no counts of drawing in pre- or post-therapy samples. However, therapy did have a significant effect on Pamela’s use of test questions: Poisson trend for frequencies (1-tailed), test questions (*z* = −6.18, *p* < .0001). Thus, for Pamela (as for Alex), behaviour change as a result of therapy constituted the reduction of a barrier conversation behaviour (in fact, test questions were almost eradicated), but there was no corresponding increase in her three chosen strategies; these remained relatively stable as the means per sample before and after therapy illustrate. In the case of Stuart, therapy had no statistically significant effect on his chosen strategies; however, numerically, keywords showed a slight increase, from a pre-therapy mean of 2.5 per sample to a post-therapy mean of 3.8 per sample. In contrast, gesture showed a numerical decrease (pre-therapy mean = 7.5, post-therapy mean = 5.2) and writing remained relatively stable (pre-therapy mean = 1.7, post-therapy mean = 1.3). All Stuart’s chosen strategies were in use before the therapy began.

In summary, after therapy, Alex and Pamela (the CPs) almost eradicated test questions from their talk; however, use of chosen strategies remained at pre-therapy levels. Whilst Graham used significantly more of his chosen strategies (writing, mime, keyword), Stuart’s use of strategies (writing, gesture, keyword) showed no significant change compared with pre-therapy levels (a numerical increase in keywords did not reach significance).

### Qualitative analysis: the impact of barriers and strategies in conversation

To shed some light on these results, it is helpful to perform a qualitative exploration of the data using CA. The analysis that follows is divided into two sections, the first explores the form and impact of test question sequences in pre-therapy conversations, the second explores the opportunity for PWA strategy use when such questioning by the CP is reduced.

#### Test questions: PWA turns highly constrained by prior sequential context

Test questions are typically a feature of pedagogic interactions, and they set up a context in which the addressee is expected to provide a very specific known answer, commonly a noun or a noun phrase. This clearly sets up a difficult interactional task for any PWA, given the persistent word finding difficulties associated with all types of aphasia. The literature reveals the social and emotional impact of test questions in aphasic conversation to be variable. Sometimes they can be a positive method of promoting language use (Aaltonen & Laakso, 2010; Bauer & Kulke, 2004) and, in other instances, a behaviour that engenders a threat to face, arouses frustration and even distress (Burch et al., 2002; Lock et al., 2001). See Beeke et al. (2013) for more detailed discussion of the interactional motivations behind test question sequences.

Extracts 1 and 2 have been chosen to illustrate the form that test question sequences commonly took in pre-therapy talk for these two dyads and to highlight their impact on the turns of Graham and Stuart, respectively.

**Table:** Extract 1: D4C2i Anne’s birthday Alex and Graham decide they will talk about what they have been doing during the week by going through the diary, which Alex is holding. After Alex mentions doing some gardening, Graham says a friend’s name (line 01).

01	Graham	erm (1.2) Anne?
02	Alex	mhm (.) for her what
03	Graham	birthday
04	Alex	yeah (0.4) what did you think of that
05	Graham	⌈(2.8) ⌉ yeah, mm (1.2) uh⌊*((Graham grimaces))* ⌋
06		⌈no⌊*((Graham grimaces, small head shake))*
07	Alex	what didn’t you like
08	Graham	(2.5) erm (5.8)
09	Alex	you went in (0.5) yeah? (0.3) sat down
10	Graham	yeah
11	Alex	and had a ⌈(0.8)⌊*((Alex gestures drinking))*
12	Graham	(2 syllables)
13	Alex	yeah
14	Graham	um
15	Alex	but what- what was difficult about the night
16	Graham	(6.3) (sighs) erm huhhhh (1.8)
17	Alex	⌈the ⌈nuh-⌊*((Alex taps his ear))*
18	Graham	⌊(and=em) (4.2) people and eh driving?
19	Alex	uhuh
20	Graham	and (2 syllables) (party)
21	Alex	mm
22	Graham	(but) eh=and ⌈bay ay ay ay ay- oh ⌉ god!⌊*((Graham raises hand to head height, gestures talking))*⌋
23	Alex	(he) was (going) (1 syllable)
24	Graham	yeah and uh deh (1.8) erm (3.3)
25	Alex	because ⌈(0.2)⌊*((Alex touches his ear))*
26	Graham	(cur-) (2.2)
27	Alex	to hear people was hard=
28	Graham	=yeah
29		(0.3)
30	Graham	yeah

In this sequence about Graham’s opinion of a birthday party they attended, Alex uses three test questions to scaffold Graham’s participation in the discussion. The first is seen in line 02, where Alex asks Graham to say why they saw Anne (“for her what”); since Alex and Graham both participated in the event involving Anne, we surmise that Alex knows the answer to his question. Graham’s response is highly constrained by the test question formulation—it must consist of a noun to stand in the place of the word “what” in Alex’s question. Graham produces “birthday”, and Alex’s evaluation of this as an acceptable answer (“yeah”, line 04) provides further evidence that this is a “test” sequence. Then Alex makes a genuine request for Graham’s opinion about the birthday party (“what did you think of that”, line 04), but as the talk progresses, Alex’s subsequent turns suggest that he already knows that Graham did not enjoy it, and he may also know why. Although it is difficult to know if “what didn’t you like” (line 07) is a genuine question, “what was difficult about the night” (line 15) appears to be designed to prompt mention of a specific negative issue already known to them both. This is suggested by Alex’s verbal and gestural cueing behaviour in line 17; he says “the nuh” while touching his ear. At this point in the sequence, his turn could be designed to offer a guess, marked with rising intonation, for Grahame to accept or reject, in order to help move the conversation along. However, this is not what Alex does; he provides a cue to elicit a specific word, “noise”, in answer to his question. Graham encounters severe difficulty when attempting to answer these questions. His responses consist of fillers and long pauses (lines 08 and 16), and his turns remain incomplete. He does appear to have some success responding to Alex’s prompt in line 11, a gestural cue to name a drink, producing syllables unintelligible to the analyst (line 12) but apparently understood by Alex, who responds “yeah” (line 13). Having ignored Alex’s cue for the word “noise” in line 17, Graham goes on to mention other things, some of them unintelligible (line 20) before describing the noise in his own way, by gesturing “chatter” and mimicking the sound of talking (“(but) eh = and bay ay ay ay ay- oh god!”, line 22). Alex appears to attempt one further elicitation of the reason why the night out was difficult, saying “because…” (line 25) and repeating the gesture of touching his ear that was seen in line 17, but Graham is unable to respond. In the end, it is Alex who voices the reason, saying “to hear people was hard” (line 27), to which Graham responds “yeah…yeah” (lines 28 and 30). This sequence is representative of Alex and Graham’s pre-therapy exchanges. It is notable that Graham has severe difficulty in producing answers constrained by test questions, yet does appear to be able to talk more freely when not required to produce such specific turns; compare direct attempts to answer test questions in lines 08 and 16 with turns in lines 18, 20, and 22. Although the impact of test questions on Graham’s turns appears problematic, such sequences are often collaborative, and there is little sign of negative emotion. Interestingly, when asked in therapy session 5 how he feels about Alex asking test questions, Graham rolls his eyes and shows quite plainly that it annoys him.

However, some people with aphasia, like Stuart, appear in their conversations to be frustrated and upset by being asked test questions. Extract 2 has been chosen to illustrate the common pattern of test questions in Stuart’s and Pamela’s conversations before therapy. In this extract, their 12-year-old son, Graham, is present.

**Table:** Extract 2: D8C7 where did you go this morning Pamela, Stuart and their 12 year old son Graham are eating around the table. Prior to this exchange, Stuart started singing and was told to talk instead.

01	Pamela	where did you go this morning?
02	Stuart	⌈(0.6) ⌉⌊*((Stuart looks at Pamela))* ⌋
03	Graham	don’t panic
04	Stuart	⌈(0.8) ⌉⌊*((Stuart nods at Graham))* ⌋
05	Pamela	Mr Mainwaring
06	Graham	hehe=
07	Pamela	=hehe=
08	Graham	=you always say that
09	Stuart	⌈(3.0) ⌉ ⌈right ⌉⌊*((Stuart opens and closes mouth))* ⌋ ⌊*((points upwards then puts hand on hip))* ⌋
10		⌈(3.4) ⌉ yep⌊*((looks into space, opens and closes mouth))* ⌋
11	Pamela	where did you go this morning?
12	Stuart	⌈(3.1) ⌉⌊*((Stuart skywrites letter W))* ⌋
13	Graham	oh you can hear the fair
14	Stuart	⌈(3.4) ⌉⌊*((Stuart puts hand on hip and looks at Graham in annoyance))* ⌋
15		⌈(sedskai) gardens ⌉⌊*((looks at Pamela))* ⌋
16	Pamela	(0.7) where did you go though?
17	Stuart	⌈(3.2) ⌉⌊*((Stuart looks down and puts arm heavily on table))* ⌋
18	Pamela	⌈where did you go ⌉ and you had a cup of tea, where did you go=⌊*((Stuart sighs heavily))* ⌋
19	Stuart	=⌈yeah ⌉⌊*((Stuart scratches head and looks away))* ⌋
20		⌈(9.2) ⌉⌊*((Stuart continues to look away, then down at table, holds head))* ⌋
21	Pamela	do you know?
22		⌈(6.1) ⌉⌊*((Stuart eats a mouthful of food, then looks at Pamela))* ⌋
23	Pamela	try and think of it while you
24	Stuart	yeah
25	Pamela	you went over thu-
26	Stuart	⌈(3.1) ⌉⌊*((Stuart looks into space, then at Pamela))* ⌋
27	Graham	the
28	Pamela	don’t give him ⌈any anymore clues because it’s cheating then
29	Stuart	⌊park?
30	Pamela	you didn’t get that because we gave you a clue

This extract opens with Pamela asking a question, “where did you go this morning?”. As the sequence progresses, it becomes clear that this something she already knows and that Stuart is required to say the “correct” word rather than talk about his day. The son’s comment “don’t panic” (line 03) reinforces the sense that Stuart is being tested and leads to a humorous aside between mother and son, centred on a catchphrase from a television programme (“don’t panic Mr Mainwaring”), while they wait for Stuart to respond (lines 03–08). Stuart recognises that an answer is required; lines 09 and 10 show signs of word search behaviour including pausing, mouth movements, gazing to the middle distance and fillers. Pamela then repeats her question, this time stressing the word “go”, marked by underlining in the transcript (line 11). Stuart writes the letter W in the air (line 12), but this strategy goes unnoticed, and Graham comments about being able to hear the fair outside their house. Stuart’s body language suggests a negative reaction to this interruption (line 14). In the next turn, Stuart manages to give a spoken but only partially intelligible response (“(sedskai) gardens”, line 15). However, Pamela’s response (“where did you go, though”, line 16), delivered after a 0.7 s pause, appears to indicate that it is not the answer she is looking for. Stuart looks down, drops his arm onto the table top and sighs heavily. Pamela asks again, and Stuart continues to have severe difficulty formulating an answer (lines 19–20). There is a 9.2 s pause, at the end of which he holds his head in his hand. It is unclear whether he will make any further attempt to respond. Recognising this, Pamela gives him the option to stop, asking “do you know?” (line 21), and Stuart resumes eating. She then encourages him to think while he eats. Next, she attempts to cue him by starting a turn for him to complete with a noun, “you went over thu-” (line 25). A 3.1 s pause follows during which Stuart gazes to the middle distance and then at Pamela. In response, Graham produces the determiner in full, saying “the” (line 27). Pamela’s reaction is to tell her son not to “give any more clues because it’s cheating then” (line 28), making explicit the interactional “rules” that are in play during this test question sequence. In overlap, Stuart tentatively offers the word “park?” (line 29). Pamela’s turn in line 30, “you didn’t get that because we gave you a clue”, suggests that Stuart has now produced the required response however, and this is not acknowledged directly; she voices a view that the “test” was not valid because they gave him a clue.

As Extracts 1 and 2 show, test question sequences constrain Graham’s and Stuart’s turn constructions; they are required to answer questions typically by producing nouns, and as a result, their turns display severe word search behaviours and often remain incomplete. After therapy, the quantitative findings reveal a significant decrease in both CPs’ test questions. This change leads to less constraint on what type of turn the PWA has an opportunity to produce, and the next section explores what effect this opening up of the turn space has on Graham’s and Stuart’s turns.

#### An opportunity for PWA strategy use when the turn space is opened up

Extracts 3 and 4 have been chosen as representative examples of post-therapy sequences of talk, where Graham and Stuart can be seen to construct turns that reach completion and are much less problematic to produce than those seen in Extracts 1 and 2. These turns are produced in the context of Alex and Pamela taking types of turns other than test questions.

**Table:** Extract 3: D4C17 Lauren Graham and Alex are sitting at a table. Graham has the diary and a notepad and pen in front of him, from which he has torn out a page which appears to be a list of things to do. They are discussing things on the list.

01	Alex	⌈and what else is on there⌊*((looking over at and pointing to an item on Graham’s list))*
02	Graham	(1.2) si- si- ⌈(susums)⌊*((points at same item on list))*
03	Alex	oh yeah cos we have to- (0.3) give them identification about- for you
04	Graham	yeah erm ⌈(3.5)⌊*((Graham looks down at list))*
05		⌈(14.5)∣*((Graham picks up pen, writes, when finished puts down pen and looks at Alex))*⌊*((Alex watches Graham write))*
06	Alex	is that ⌈London⌊*((looks up from watching what Graham has written))*
07	Graham	⌈(lunuh)⌊*((Graham points to what he has written))*
08	Alex	Lauren
09	Graham	yeah
10	Alex	for the will as well
11	Graham	yeah
12	Alex	oh yeah
13		(1.0)
14	Alex	(if y-) phone her u- WELL we should dig it out (0.3)
15	Alex	⌈and then make the ch- copy it make ⌈changes⌉ (.) ⌊*((Alex mimes writing in the air))*⌋ ⌊*((Graham nods))*
16		I’m happy to take in=she might just do it by post
17		(0.3)
18	Graham	mm
19	Alex	but we’ll need to like work out who (.) what and all that (0.8) beforehand
20		(5.3)
21	Alex	did you want that done before we go away
22	Graham	yeah
23	Alex	⌈(0.4) sensible ⌊*((nods))*

In this extract, Graham and Alex are discussing an item on their “to do” list, which possibly relates to legal issues (identification documents, Graham’s will); although it is not entirely clear what is being talked about (in part because Graham’s identification of a key referent is distorted by dyspraxia), the speakers appear to understand each other fully. When asked by Alex about an item on the list, Graham verbalises a name that sounds like “susums” (line 02). Alex’s response is quick and makes apparent his understanding not only of the referent but also of what has to be done “…we have to give them identification…for you” (line 03). Graham agrees and then begins a word search, saying “erm” and looking down at his list for 3.5 s. Subsequently, he picks up a pen and begins writing for 14.5 s (line 05), during which time Alex watches. When Graham puts the pen down, Alex asks for clarification of what he has written, saying “is that London” (line 06). In response, Graham offers what appears to be a correction (“(lunuh)”, line 07); his production of the word is distorted by dyspraxia but Alex understands it to be “Lauren” (line 08), which Graham accepts. Alex then shows his understanding of how this referent links to the topic of talk, saying “for the will as well” (line 10). They then go on to have a discussion about how best to change the will, and when. By using his chosen strategy of writing, Graham is able to circumvent his severe word finding difficulties to take a turn that extends the current topic and launches discussion about an issue of importance to him. It is notable that before therapy, Graham did not write to circumvent his word finding problems—there were no examples of writing in pre-therapy samples (see Table 2). In addition, this extract shows how Alex’s turns differ from pre-therapy test questions. Here he is commenting and offering his opinion (lines 03, 10, 14–16, 19, and 23), and when he does ask a question, it is one to which he genuinely does not appear to know the answer (“did you want that done before we go away”, line 21). Arguably, Alex has the option to take turns like this precisely because Graham has been able to influence the conversation by introducing new referents and generally taking a more active role. The quantitative findings reveal no significant change in Alex’s use of his chosen strategies (one of which was commenting), which implies that he is doing no more commenting than he was before therapy. And yet he is using significantly fewer test questions. Qualitatively, it appears that he is commenting, offering his opinion and asking genuine questions.

**Table:** Extract 4: D8C13 mini convertible Pamela, Stuart and their 12-year-old son Graham are talking about Stuart’s outing with his friend Sonny earlier in the day.

01	Pamela	did Sonny have his mini?
02	Stuart	yeah
03	Pamela	he took him in a mini convertible *[said to Graham]*
04	Stuart	yeah HAHAHAHAHAHAHAH
05	Pamela	ohhhh you poser you!
06	Stuart	⌈yes indeed!⌊*((salutes))*
07	Pamela	where was you in the front?
08	Stuart	well er d’you know what I’m saying
09	Pamela	where was you sitting
10	Stuart	yeah yeah
11	Pamela	in the front
12	Stuart	yeah
*[5 lines between Graham and Pamela about who owns the car omitted]*		
13	Pamela	and Jane was in the back
14	Stuart	yeah
15		(2.0)
16	Pamela	I don’t know
17	Stuart	I don’t know
*[10 lines about a phone call between Pamela and Sonny omitted]*		
18	Pamela	oh so you was posing ⌈were you
19	Stuart	⌊I know
20	Pamela	eh? (.) in your (.) in his mini convertible=what- I’ve forgotten what colour it is
21	Stuart	yeah erm (0.5) ⌈blue,
22	Graham	⌊is it white?
23	Stuart	⌈eh no, ⌉⌊*((Stuart holds hand up))*⌋
24	Graham	⌈because normally th- there’s mini- white mini⌊*((Stuart puts hand on hip, looks down at table top))*
25		con⌈vertibles
26	Stuart	∣WHITE!⌊*((Stuart looks up at Pamela and sky-writes letter W))*
27		⌈erm ⌉ (0.3) white a⌈nd⌊*((moves index finger up and down in the air))*
28	Pamela	⌊oh it’s white ⌋
29	Graham	⌊green
30	Stuart	⌈no ⌉⌊*((Stuart shakes head))*⌋
31	Pamela	⌈Graham! let him say⌈it himself⌊*((Stuart moves hand in dismissive action, looks down at table, drops hand))*
32	Graham	⌊don’t worry I’m not gonna-
33	Stuart	⌈(0.2) ⌈black ⌈(.) black=⌊*((Stuart raises arm))*⌊*((sky-writes letter B))*
34	Graham	⌊black
35	Pamela	=(what) black and ⌈white! ⌉
36	Stuart	⌊*((Stuart does thumbs up to Pamela))*⌋ exactly

In this extract Pamela asks Stuart about his trip out with Sonny earlier that day. Although Pamela still asks Stuart questions, there is a marked contrast with pre-therapy talk as exemplified in Extract 2. Rather than prompting Stuart to name referents that are already shared knowledge (where he went and with whom), Pamela instead asks about details that she does not appear to know, such as what car they went in and where Stuart sat. Importantly, she also takes different kinds of turns to those seen pre-therapy, creating humour with “ohh you poser you!” (line 05) and “I don’t know” (line 16), said with mock despair. By constructing turns that express her opinions, rather than asking test questions, she succeeds in collaborating with Stuart to create a light-hearted exchange, which both of them appear to enjoy. Thus, although the quantitative findings reveal no significant change in Pamela’s use of her chosen strategies, there appear to be qualitative changes in her post-therapy turn types. In line 20, she asks Stuart to tell her what colour the car is, a question that bears a resemblance to those seen in their pre-therapy conversations. Interestingly, she begins a direct question, saying “what”, but immediately reformulates it into a statement that focuses on her own failings, “I’ve forgotten what colour it is”. The fact that we cannot tell from the sequence of turns if she knows the colour of the car, and thus whether this is a test question, is testament to the subtlety of her interactional behaviour here. Her question casts Stuart in the role of knowing the answer, rather than being “tested”. One final turn of interest is line 31 where Pamela asks their son to stop interrupting Stuart. This suggests a shift from pre-therapy conversation dynamics in which Pamela and Graham collaboratively prompted Stuart to talk. For his part, Stuart succeeds in providing the answer over lines 21–36, in collaboration with Pamela and Graham. Some of Stuart’s turns appear to be constructed using similar strategies as those used before therapy, namely keywords and writing letters in the air. However, in this post-therapy extract, Stuart makes more effective use of these strategies, in part because Pamela appears to treat his sky writing as part of his turn (compare this with Extract 2 line 12 where sky writing is ignored). Thus, he perseveres with a self repair to successfully identify the car as “white and… black” (lines 27 and 33). In addition, Stuart takes very different kinds of turns to those seen in Extract 2. He deploys stereotyped phrases such as “yes indeed” (line 06), “d’you know what I’m saying” (line 08), “I don’t know” (line 17) and “I know” (line 19) to great conversational effect, allowing him to collaborate with Pamela to create a humorous story about his day out.

## Discussion and clinical implications

This article reports the quantitative and qualitative therapy outcomes of conversation training for two dyads where one speaker has agrammatic aphasia. The quantitative results reveal that therapy had a significant effect on both speakers of one dyad, Alex (CP) and Graham (PWA), but only on the CP of the other dyad, Pamela, and not on Stuart (PWA). Thus, Alex almost eradicated test questions from his talk after therapy, having identified this as a barrier behaviour that he wished to change. Qualitative analysis showed that Alex was taking facilitative turn types (such as commenting and asking genuine questions) after therapy; however, the quantitative analysis revealed no significant increase in the specific strategies that he chose to target (“let the conversation continue”, “carry on if you have understood”, “comment”); commenting was present in pre-therapy samples. Graham, his CP with aphasia, used significantly more of the strategies he chose to practise during therapy (writing, mime, keywords).

While Pamela too almost eradicated test questions from her talk after therapy, she did not significantly increase her use of chosen strategies (“let the conversation continue”, “carry on if you have understood”, “paraphrase”), though qualitative changes were noted. Post-therapy turns expressed her opinions, and questions appeared to arise from a genuine lack of knowledge. There was no significant change in strategy use (writing, gesture, keyword) for her CP with aphasia, Stuart, although a numerical increase in keywords was noted. These findings strengthen the evidence base for the success of conversation therapy in changing the behaviours of CPs and also add quantitative evidence to the growing case for the effectiveness of direct conversation training for some people with aphasia.

With both CPs asking significantly fewer test questions, the post-therapy conversational context for both PWAs changed. They were presented with an opportunity to take turns that were less constrained; they were no longer frequently being selected by the CP to produce a specific (and often known) answer. In this relatively open post-therapy turn space, both increased their use of keywords, but this only reached statistical significance for Graham. Graham also increased his use of writing and mime, yet Stuart showed no change in his use of writing and, indeed numerically, gestures reduced. So we might ask why Stuart was less able to take advantage of the changed interactional context in his conversations with Pamela and was less able to deploy the strategies he practised during therapy. One reason appears to be his pattern of language impairments. As Figure 3 shows, Stuart scored zero on the PALPA written picture naming test (Kay, Lesser, & Coltheart, 1992). (Graham scored 13/30 on the same test.) In conversation, we see Stuart successfully writing the first letter of a word in the air (see Extracts 2 and 4), but he does not attempt to spell out more of the word and never resorts to pen and paper. So it appears that, for Stuart, the potential of writing as a strategy to aid conversation is limited by his aphasia. However, for Graham, this strategy appears much more functional, as reflected in his increased use of writing after therapy and the success with which he and Alex collaborate to make use of it in their conversations. It is less clear why Stuart appeared to be using fewer gestures after therapy, despite choosing this as another of his strategies to practise. It might be that, before therapy, when required to produce a specific, concrete noun in response to a test question, Stuart was facilitated to produce a gesture instead of or alongside the lexical item required in his answer. After therapy, it seems possible that the opportunity to take a range of different turn types may have adversely influenced gesture use. With impaired non-verbal semantics—he scored 44/52 on the Pyramids and Palmtrees Test (Howard & Patterson, 1992)—the semantic constraint imposed by a test question may have aided his production of gesture. Qualitative analysis of the context of gesture use in conversations before and after therapy is required to further explore this idea.

If, as it appears, the extent and pattern of language deficit has an impact on a PWA’s ability to learn to deploy conversational strategies successfully, we may need to reconsider the conversation therapy goal setting process. When working with a CP on conversation training, it is common for the SLT to facilitate the CP to choose his or her own strategies, as it is felt that this has a beneficial effect on motivation to change behaviour. However, transferring this goal setting technique to direct work with a PWA appears to raise additional challenges, in that he or she may choose a strategy that has limited use, given his or her aphasic deficits.

It is interesting to note that, although both CPs reduced their use of test questions, neither significantly increased the use of their strategies chosen to enhance conversation. For Pamela, qualitative analysis revealed the use of different types of turns after therapy (genuine questions, opinions), whereas Alex continued to comment, as he had been doing before therapy. Therapy aimed to reduce barrier behaviours such as test questions indirectly: it was hypothesised that such barriers would fall away as other positive conversational behaviours came online. However, this does not appear to be the mechanism for behaviour change that operated for Alex or for Pamela. Intuitively, it seems possible that stopping oneself from engaging in negative behaviours might be easier than learning to systematically use positive ones (consider not eating chocolate versus remembering to incorporate more fibre into your diet, for example). Perhaps a single, very distinctive, conversational barrier can be avoided with relative ease once one has insight into its negative effects, yet more complex behaviour change is required to deploy a facilitative strategy in an appropriate interactional context. This idea clearly has important implications for how SLTs might guide the goal setting process and deliver conversation therapy. Research into behaviour change mechanisms in conversation therapy is currently under way by the authors, led by Johnson.

Finally, it is recognised that stability of interactional behaviours may vary naturally, both within a conversational sample and over time (Perkins, Crisp, & Walshaw, 1999). This study has attempted to mitigate for within-participant variation by measuring behaviours across multiple baselines as advised by Perkins et al. (1999), analysing six pre- and six post-therapy 5 min conversation samples in total. The extensive sampling of conversation provides a rich source to analyse for quantitative and qualitative explanations of behaviour change after conversation therapy, and as our analysis expands to include additional dyads, the number of repeat recordings will continue to help us answer questions about stability of strategies used.

## Conclusions

In this article, quantitative and qualitative analyses of pre- and post-therapy conversations between two dyads have been presented and discussed to illustrate not only whether but *how* conversation therapy might achieve behaviour change in a CP and in a PWA. Both dyads show evidence that collaborative work between an SLT, PWA, and CP can produce tangible improvement in conversational exchanges in the chronic stage of aphasia. Certain aspects of conversation (both positive and negative) captured by video were highlighted by the SLT for discussion, then the dyads selected and practised strategies to enhance their communication. A quantitative analysis of barriers and strategies across pre- and post-therapy conversation samples, combined with a qualitative analysis of interaction, revealed that the same change in conversational behaviour of two CPs (significant reduction in test questions) went hand in hand with a different outcome for each CP’s respective PWA; the combination of strategies that work for one dyad may not work for another. Questions have been raised about what types of behaviour may be more amenable to change and the effect of a PWA’s language deficit. Once the data from all eight dyads have been analysed, conclusions about the effectiveness of this intervention will be reconsidered in the light of variation of language profiles and other variables recorded in this study.

## Funding

This work was funded by a Stroke Association project grant [grant number TSA 2007/05, 2008–2011] and data are lodged in the human Communication Audio Visual Archive (CAVA) at UCL (www.ucl.ac.uk/ls/cava/). We are very grateful to Graham and Alex, and Stuart and Pamela (pseudonyms) for their enthusiastic participation and commitment to the project and to their respective SLTs for referring them to us. *Better Conversations with Aphasia*, a free e-learning resource available at UCLeXtend, was funded by the Economic and Social Research Council [grant number RES-189-25-0292]. The data analysis presented in this article builds on and extends the work of six Masters students of speech and language therapy at the University College London, who undertook their final year dissertations with the authors: Selina Buenaventura, Fran Children, Fiona Jorrisch, Sarah Lambert, Kate Middleton, and Amie Wilson. We thank them for their work on the ratings reported here. The first author has refined the CA transcriptions produced by Sarah Lambert (Extract 2), Selina Buenaventura (Extract 3), and Kate Middleton (Extract 4) and has extended their analyses of these data for use in this article. The transcript and analysis of Extract 1 is the first author’s own work.
